# Supplementary material for: Co-evaporation as an optimal technique towards compact methylammonium bismuth iodide layers
Source: Sci Rep. 2020 Jun 30;10:10640. doi: 10.1038/s41598-020-67606-1 (PMC7327053; doi:10.1038/s41598-020-67606-1)
Supplement: Supplementary file 1 — Supplementary information [file 41598_2020_67606_MOESM1_ESM.docx]

**Supplementary Information**

Co-evaporation as an optimal technique towards compact methylammonium bismuth iodide layers

Cristina Momblona,^1^ Hiroyuki Kanda,^1^ Albertus Adrian Sutanto,^1^ Mounir Mensi,^1^ Cristina Roldán-Carmona,^1,*^ Mohammad Khaja Nazeeruddin.^1,*^

AFFILIATIONS

^1^Group for Molecular Engineering of Functional Materials, Institute of Chemical Sciences and Engineering, EPFL Valais Wallis, Rue de l’Industrie 17, CH-1951 Sion, Switzerland.

*Email: cristina.roldancarmona@epfl.ch, mdkhaja.nazeeruddin@epfl.ch

**Table S1**. MAI and BiI_3_ deposition rates used for co–evaporation of MBI films **1** – **5**.

| Deposition rate (Å s^–1^) | Condition/Film | | | | |
| --- | --- | --- | --- | --- | --- |
|  | **1** | **2** | **3** | **4** | **5** |
| MAI | 0.4 | 0.55 | 0.7 | 1 | 1.4 |
| BiI_3_ | 0.4 | 0.4 | 0.4 | 0.4 | 0.4 |

**Figure S1**. XRD patterns of layer **1** measured as-deposited and after 4 months stored in dark at room temperature and relative humidity of 10%.

**Figure S2**. XRD patterns of layer **2** measured as-deposited and after 4 months stored in dark at room temperature and relative humidity of 10%.

**Figure S3**. XRD patterns of layer **5** measured as-deposited and after 4 months stored in dark at room temperature and relative humidity of 10%.

**Figure S4. a)** UPS spectra of MBI layer **4** and **b)** schematic energy level diagram of MBI extracted from UPS measurements and optical band gap.


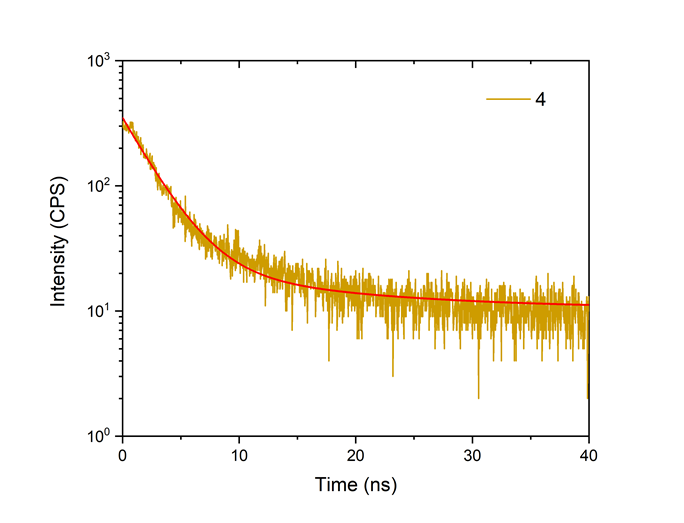


**Figure S5**. Time-resolved photoluminescence decay for MBI film nr. **4** proved at 575 nm upon excitation at 455 nm.


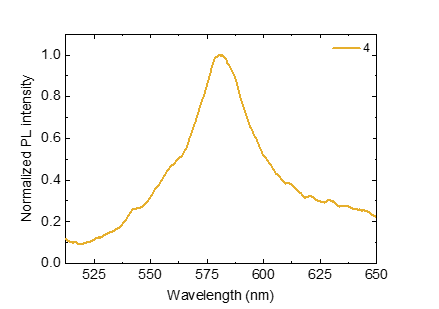


**Figure S6**. Photoluminescence spectra of MBI film nr. **4** deposited on glass upon excitation at 450 nm.

**Figure S7**. EQE of *n*-*i*-*p* solar cells with 250 nm thick MBI film.
